# Nicotinic modulation of cortical circuits

**DOI:** 10.3389/fncir.2014.00030

**Published:** 2014-03-28

**Authors:** Sergio Arroyo, Corbett Bennett, Shaul Hestrin

**Affiliations:** Department of Comparative Medicine, Stanford University School of MedicineStanford, CA, USA

**Keywords:** cholinergic, nicotinic receptors, interneuron, volume transmission, optogenetics

## Abstract

The ascending cholinergic neuromodulatory system sends projections throughout cortex and has been shown to play an important role in a number of cognitive functions including arousal, working memory, and attention. However, despite a wealth of behavioral and anatomical data, understanding how cholinergic synapses modulate cortical function has been limited by the inability to selectively activate cholinergic axons. Now, with the development of optogenetic tools and cell-type specific Cre-driver mouse lines, it has become possible to stimulate cholinergic axons from the basal forebrain (BF) and probe cholinergic synapses in the cortex for the first time. Here we review recent work studying the cell-type specificity of nicotinic signaling in the cortex, synaptic mechanisms mediating cholinergic transmission, and the potential functional role of nicotinic modulation.

## Introduction

Cholinergic axons from the basal forebrain (BF) innervate the entire cortex and are the main source of cortical acetylcholine (ACh; Mesulam et al., 1983; Rieck and Carey, 1984; Rye et al., 1984; Saper, 1984; Eckenstein et al., 1988). Endogenously released ACh activates both metabotropic muscarinic and/or ionotropic nicotinic acetylcholine receptors (nAChRs) expressed on cortical neurons. In this review, we will focus on nAChR activation in the cortex.

Nicotinic receptors are pentameric proteins comprised of particular combinations of subunits α2–α7 and β2–β4 (Cordero-Erausquin et al., 2000; Dani and Bertrand, 2007). In the cortex, two main types of nAChRs predominate: the low affinity homomeric α7 receptor and the high affinity heteromeric α4β2 receptor, though the α5 subunit is expressed to a lesser extent as well (Winzer-Serhan and Leslie, 2005; Kassam et al., 2008). Because these receptors exhibit distinct cationic permeabilities, agonist affinities, and desensitization properties (Dani and Bertrand, 2007), phasic activation of cholinergic BF axons can produce a temporally complex pattern of nAChR-dependent activation in cortical neurons depending on the identity and proportion of receptor subtypes being expressed.

## Cell-type specificity of nicotinic receptor expression

**Figure 1:**
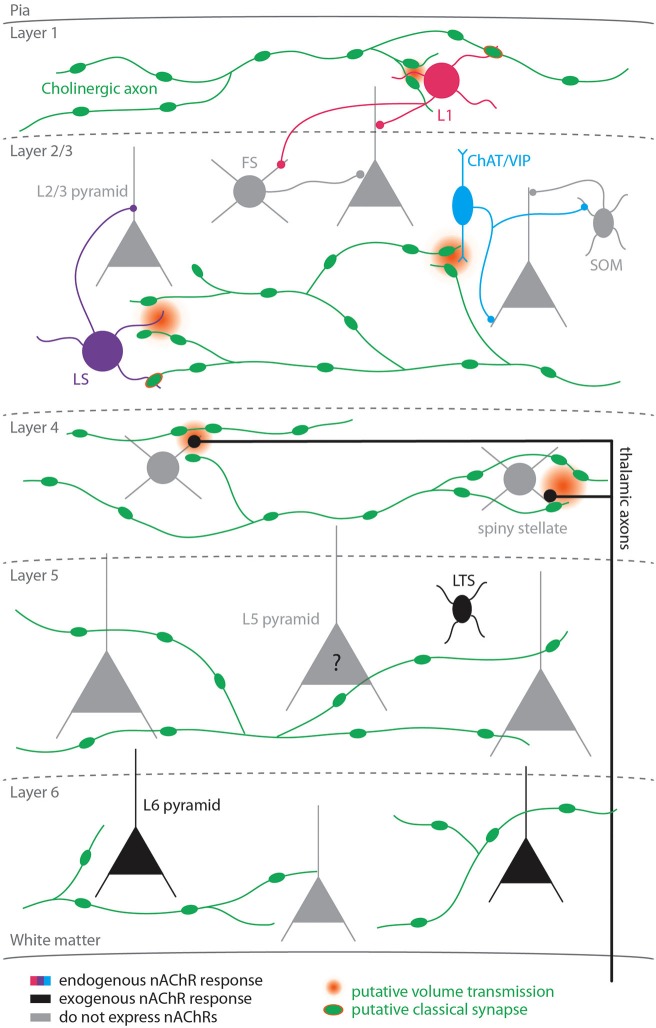
** Nicotinic signaling in the cortex**. Colored cells represent cell-types known to exhibit nAChR-dependent responses to activation of cholinergic axons; black cells represent cell-types that exhibit nicotinic responses to exogenous application of cholinergic agonists; gray cells represent cell-types that do not express nicotinic receptors. The question mark for L5 pyramidal cells reflects the fact that studies disagree as to whether this cell-type expresses functional nicotinic receptors. Two types of nicotinic signaling are depicted: putative volume transmission targeting non-α7 nAChRs (gradient) and putative classical synapses targeting α7 nAChRs (green symbol with orange border).

Several studies applying exogenous cholinergic agonists have demonstrated that only a fraction of cortical cells express functional nAChRs (summarized in Figure 1). In the supragranular layers, nicotinic receptors are expressed exclusively in inhibitory cells, including all L1 interneurons (Christophe et al., 2002; Gulledge et al., 2007) and a heterogeneous subset of L2/3 interneurons that co-express one or more of the following biochemical markers: vasoactive intestinal peptide (VIP), cholecystokinin, calretinin, calbindin, and neuropeptide Y (Porter et al., 1999; Gulledge et al., 2007). However, in two of the most prominent classes of inhibitory cells, parvalbumin (PV)-expressing and somatostatin (SOM)-expressing interneurons, nAChR expression is either absent or sparse (Porter et al., 1999; Gulledge et al., 2007). Interestingly, many if not all nAChR-expressing interneurons also express the ionotropic serotonergic receptor (5HT3, Férézou et al., 2002; Lee et al., 2010). Given that cholinergic cells in the BF and serotonergic cells in the raphe nucleus are both more active during wakefulness than during non-rapid eye movement sleep (Wu et al., 2004; Lee et al., 2005), the cortical targets on which these neuromodulatory systems converge may play a role in producing the pattern of activity associated with wakefulness.

Less is known about the pattern of nAChR expression in the lower cortical layers. Nicotinic receptors are expressed presynaptically on thalamocortical axons in L4 (Gil et al., 1997; Disney et al., 2007) where they have been shown to enhance sensory responses (Disney et al., 2007). In L5, nicotinic responses have been reported in low-threshold spiking (LTS; Xiang et al., 1998; but see Porter et al. (1999); Gulledge et al. (2007)) but not fast-spiking (FS) interneurons (Xiang et al., 1998; Porter et al., 1999; Gulledge et al., 2007). Thus, in both supra- and infragranular cortex PV+ interneurons do not exhibit postsynaptic nicotinic responses, suggesting that some rules for nAChR expression in GABAergic cells may be shared between the upper and lower layers (Gulledge et al., 2007). Interestingly, in contrast to pyramidal cells in the supragranular layers, nicotinic responses have been demonstrated in L6 pyramidal neurons (Kassam et al., 2008) and L5 pyramidal neurons (Zolles et al., 2009; Poorthuis et al., 2013), although responses in L5 pyramidal neurons have not been universally reported (Porter et al., 1999; Gulledge et al., 2007).

## Basal forebrain (BF) cholinergic axons target specific cortical cell types

The properties of α7 and non-α7 receptors and their pattern of expression in cortical cells suggest that postsynaptic nicotinic responses may vary in their kinetics. In order to study the properties of nAChR-mediated responses in cortex, it is necessary to record responses to selective activation of cholinergic fibers. Several recent studies have used optogenetic tools to probe cholinergic synapses throughout the brain, including the hippocampus (Gu and Yakel, 2011), thalamus (Sun et al., 2013), interpeduncular nucleus (Ren et al., 2011), and striatum (English et al., 2012). In the cortex, we have recently shown that L1 interneurons, L2/3 late-spiking (LS) interneurons, and L2/3 choline acetyltransferase (ChAT)-expressing interneurons (a class of cells that also express VIP) exhibit nicotinic responses following photostimulation of channelrhodopsin-2 (ChR2)-expressing BF axons (Arroyo et al., 2012). The endogenous nicotinic response in L1 and L2/3 LS cells was mediated both by α7 and non-α7 nAChRs, while the responses in L2/3 ChAT/VIP-expressing cells exhibited only non-α7 receptor responses.

**Figure 2:**
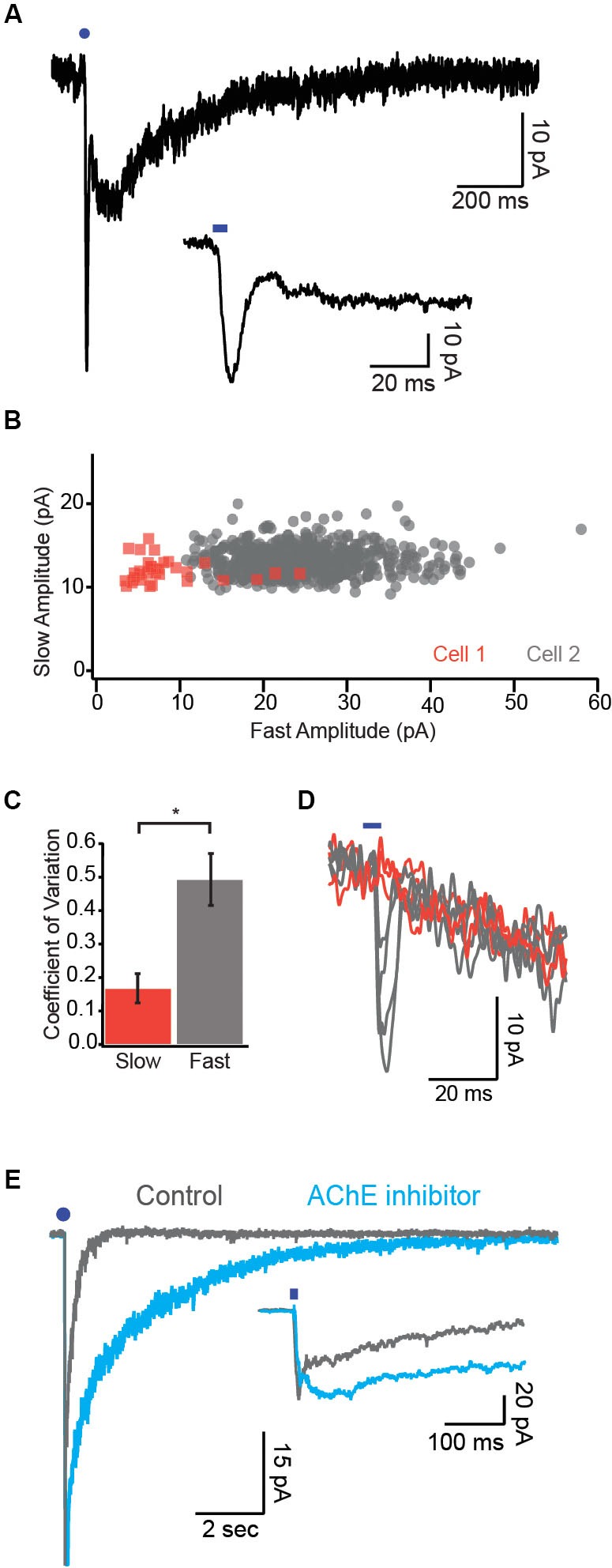
** Synaptic mechanisms underlying cholinergic transmission**. **(A)** Example dual-component response recorded under voltage clamp. Note the fast α7 mediated response followed by the slower non-α7 response. Inset, fast component is displayed on an expanded timescale. **(B)** Response amplitude for the slow component is plotted against the response amplitude of the fast component for two cells. Note that the fast component exhibits much more variability in amplitude relative to the slow component. **(C)** Variability of the two response components quantified as the coefficient of variation (CV). **(D)** Example single trial responses to photostimulation demonstrate a reliable slow component across trials in which the fast component varied widely. Orange traces represent trials in which a fast component was not detectable. **(E)** Dual-component nicotinic responses before and after application of the AChE inhibitor ambenonium. Inset, expanded timescale reveals no effect of the AChE blocker on the fast component. Blue circles and ticks represent photostimulation.

By eliciting endogenous release of ACh from BF cholinergic axons, we were able to characterize the cholinergic synapse in the cortex for the first time and identify the time course of nAChR-mediated responses. Interestingly, the kinetics of the responses mediated by α7 and non-α7 nAChRs differed by an order of magnitude (α7: rise time ~3 ms, decay tau ~5 ms; non-α7: rise time ~35 ms, decay tau ~200 ms; Figure 2A, Arroyo et al., 2012). Although the peak amplitude of the fast α7 response was often larger, more charge was transferred via the slower non-α7 response, leading to a slow barrage of disynaptic inhibition in upper layer pyramidal neurons and FS cells (Arroyo et al., 2012).

## Mechanisms underlying nicotinic transmission in the cortex

Cholinergic cells in the BF project throughout the cortex where they form a dense web of presynaptic varicosities spanning all cortical layers. Numerous anatomical studies observed that a large fraction of these varicosities are not directly adjacent to postsynaptic structures, leading to the hypothesis that the cholinergic system operates primarily by diffuse release of neurotransmitter into the extracellular space (“volume transmission”) (Mrzljak et al., 1993; Umbriaco et al., 1994; Lendvai and Vizi, 2008; Yamasaki et al., 2010), though others have emphasized the presence of classical synaptic contacts (Turrini et al., 2001).

The presence of both a slow nicotinic response mediated by the high affinity non-α7 receptor and a fast response mediated by the low affinity α7 receptor (Arroyo et al., 2012) led us to hypothesize that these two response components might be mediated by volume transmission and classical synaptic transmission, respectively. We performed several lines of experiments to test this possibility.

The trial-to-trial variability of synaptic responses depends in part on the number of release sites mediating transmission between presynaptic fibers and the postsynaptic cell (Manabe et al., 1993). Because non-synaptic receptors activated by volume transmission can sample release from many presynaptic sites, this form of signaling should be characterized by low variability (Szapiro and Barbour, 2007). In cells exhibiting dual component excitatory postsynaptic currents (EPSCs; Figure 2A) we found that the response variability of the slow component was several-fold smaller than that of the fast component as quantified by the coefficient of variation (CV; Figures 2B, C, Bennett et al., 2012). Moreover, the amplitudes of the fast and slow response components were not correlated across single trials (Figure 2D). These data are consistent with the notion that the slow response component is mediated by ACh release from many non-synaptic release sites while the fast response component is mediated by relatively fewer release sites onto classical postsynaptic terminals.

Responses mediated by volume transmission are highly sensitive to perturbations of transmitter clearance (Szapiro and Barbour, 2007). We found that application of an AChE inhibitor drastically prolonged the decay of the slow but not the fast nicotinic response (Figure 2E, Bennett et al., 2012). Moreover, application of exogenous AChE selectively attenuated the slow response (Bennett et al., 2012). Together, these data suggest that the fast and slow nicotinic responses are mediated by distinct synaptic mechanisms.

A conclusive determination of synaptic or non-synaptic transmission requires detailed anatomical reconstruction of receptor localization relative to presynaptic varicosities and a characterization of the kinetics of α7 and non-α7 receptors. To date, no anatomical study has examined the spatial relationship between nicotinic receptor subtypes and cholinergic varicosities in the cortex. Furthermore, though we were able to estimate the kinetics of α7 receptors for a range of ACh concentrations using nucleated patches, we did not observe non-α7 receptor responses in this preparation, and no previous studies report the kinetics of natively expressed non-α7 receptors.

Given the lack of anatomical data, we cannot exclude the possibility that α7 receptors are located perisynaptically and not at classical postsynaptic specializations, since both of these arrangements could produce high variability and insensitivity to AChE perturbation. Similarly, our data do not definitively rule out the possibility that non-α7 receptor-mediated currents are synaptic. However, the synapse mediating this response would have to fulfill several specific criteria. To explain our AChE perturbation results, the synaptic cleft would have to be constructed such that activation of postsynaptic receptors is primarily limited by hydrolysis of ACh by AChE rather than diffusion. This is remarkable given that diffusion of neurotransmitter out of a conventional synaptic cleft is extremely fast (concentration decay *t*_1/2_ ~0.15 ms; Eccles and Jaeger, 1958). Moreover, the slow rise time of the non-α7 receptor-mediated EPSC (20–80% in 35 ms) would require that these receptors exhibit exceptionally slow activation kinetics. Since both synaptic and nonsynaptic cholinergic varicosities are found in cortex, we believe that a more parsimonious explanation of our data is that non-α7 nicotinic receptors are located extrasynaptically where they bind ACh diffusing from nonsynaptic release sites.

## Functional consequences of nicotinic receptor activation in the cortex

Numerous studies have demonstrated that activation of nicotinic receptors is critical for normal cognition. Administration of nicotine has been shown to enhance working memory and attention and to alleviate the cognitive deficits observed in multiple neuropsychiatric conditions (Levin, 2002). Furthermore, loss of the β2 nAChR subunit, a necessary component of the high affinity non-α7 cortical nAChR (α4β2), has been shown to impair both learning (assayed by a passive avoidance task; Picciotto et al., 1995) and attention (assayed by the 5 choice serial reaction time test, 5CSRTT; Cordero-Erausquin et al., 2000; Guillem et al., 2011). Though knockout of the α7 nAChR subunit does not affect gross neurological function (Orr-Urtreger et al., 1997) or performance on the 5CSRTT (Grottick and Higgins, 2000; Howe et al., 2010; Guillem et al., 2011), recent evidence suggests that activating α7 nAChRs may alleviate the cognitive impairments associated with Alzheimer’s disease and schizophrenia (Levin, 2013). Recently, it was shown that optogenetic activation of BF cholinergic axons in visual cortex enhanced performance on a visual discrimination task, while silencing BF cholinergic cells impaired performance (Pinto et al., 2013). However, whether this effect was mediated by nicotinic or muscarinic receptors was not investigated.

Several mechanisms have been proposed to account for the behavioral enhancements associated with nAChR activation. First, it has been suggested nAChR activation may lead to amplification of sensory responses by modulating release from thalamocortical terminals. Indeed, in brain slices preserving thalamocortical connections, it was shown that release from thalamocortical terminals is enhanced by nicotine (Gil et al., 1997). A recent study extended this finding by showing that iontophoresis of nicotine in primate visual cortex augments responses to visual stimuli (Disney et al., 2007). Interestingly, nAChRs are present on thalamocortical axons targeting excitatory but not inhibitory cells in L4 (Disney et al., 2007; Kruglikov and Rudy, 2008), suggesting that ACh may play a role in modulating the balance of excitation and inhibition elicited by sensory stimuli.

Another line of studies suggests that nAChR activation may shape the spatiotemporal pattern of inhibition in cortex by differentially modulating the excitability of distinct classes of interneurons. Our data demonstrate that activation of cholinergic axons in brain slices elicits disynaptic inhibition in both pyramidal neurons and inhibitory FS cells (Arroyo et al., 2012). This nAChR-dependent inhibition of FS cells is consistent with a recent study showing that cholinergic activation following foot shock inhibits spiking in L2/3 PV+ neurons in auditory cortex (Letzkus et al., 2011). In this study, the authors show that a fraction of L1 interneurons exhibit a nAChR-dependent increase in spiking after foot shock and suggest that these cells mediate the inhibition observed in PV+ cells; however, whether other nAChR expressing interneurons in L2/3 play a role in mediating cortical disinhibition was not definitively ruled out. Indeed, two recent studies suggest that another population of nAChR-expressing cells, VIP+ interneurons, preferentially target SOM-expressing interneurons in the visual cortex (Pfeffer et al., 2013) and barrel cortex (Lee et al., 2013) and, to a lesser degree, PV+ interneurons (Dávid et al., 2007; Hioki et al., 2013; Pi et al., 2013). Thus, it is likely that nAChR activation produces disinhibition via both L1 interneurons (Christophe et al., 2002; Letzkus et al., 2011; Jiang et al., 2013) and L2/3 VIP+ interneurons (Lee et al., 2013; Pfeffer et al., 2013).

The substantial difference in kinetics between α7 and non-α7 nicotinic receptors together with their cell-type specific expression suggests that these two nAChRs may play distinct roles in modulating cortical activity. For example, temporally precise excitation mediated by α7 receptors may synchronize activity in α7-receptor expressing interneurons. In contrast, slow excitation mediated by non-α7 receptors may facilitate modulatory pathways that unfold over longer time scales. Indeed, nAChR-expressing interneurons have been implicated in a number of slow processes, including inhibition mediated by postsynaptic GABA_B_ receptors (Tamás et al., 2003), reduction of synaptic efficacy by activation of presynaptic GABA_B_ receptors (Oláh et al., 2009; Chittajallu et al., 2013), and regulation of cerebral blood flow (Cauli et al., 2004).

## Future directions

Ultimately, understanding how nAChR activation modulates cortical activity will require a more complete understanding of (1) the patterns of activity in cortically projecting cholinergic axons during behavior; (2) the functional roles of nAChR-expressing cortical neurons and their subsequent modulation by endogenously released ACh; and (3) the respective impact of fast and slow nicotinic modulation on cortical circuits. The recent proliferation of Cre-driver lines has allowed investigators to begin to probe the function of various classes of cortical cells, including some cell-types known to express nAChRs. However, further work is needed to uncover how the function of these cortical neurons is modulated by activation/silencing of cholinergic fibers and blockade of specific receptor subtypes. Given the well-established role for nicotinic signaling in numerous neuropsychiatric diseases, a better understanding of the mechanisms underlying nicotinic modulation of cortical activity holds promise for the development of more effective therapeutic interventions.

## Conflict of interest statement

The authors declare that the research was conducted in the absence of any commercial or financial relationships that could be construed as a potential conflict of interest.
